# Evaluation measure for group-based record linkage

**DOI:** 10.23889/ijpds.v4i1.1127

**Published:** 2019-11-29

**Authors:** C Nanayakkara, P Christen, T Ranbaduge, E Garrett

**Affiliations:** 1 Research School of Computer Science, The Australian National University, Canberra, ACT 2601, Australia; 2 Department of History, University of Essex, Essex, United Kingdom

## Abstract

**Introduction:**

The robustness of record linkage evaluation measures is of high importance since linkage techniques are assessed based on these. However, minimal research has been conducted to evaluate the suitability of existing evaluation measures in the context of linking groups of records. Linkage quality is generally evaluated based on traditional measures such as precision and recall. As we show, these traditional evaluation measures are not suitable for evaluating groups of linked records because they evaluate the quality of individual record pairs rather than the quality of records grouped into clusters.

**Objectives:**

We set out to highlight the shortcomings of traditional evaluation measures and then propose a novel method to evaluate clustering quality in the context of group-based record linkage.

**Methods:**

The proposed linkage evaluation method assesses how well individual records have been allocated into predicted groups/clusters with respect to ground-truth data. We first identify the best representative predicted cluster for each ground-truth cluster and, based on the resulting mapping, each record in a ground-truth cluster is assigned to one of seven categories. These categories reflect how well the linkage technique assigned records into groups.

**Results:**

We empirically evaluated our proposed method using real-world data and showed that it better reflects the quality of clusters generated by three group-based record linkage techniques. We also showed that traditional measures such as precision and recall can produce ambiguous results whereas our method does not.

**Conclusions:**

The proposed evaluation method provides unambiguous results regarding the assessed group-based record linkage approaches. The method comprises of seven categories which reflect how each record was predicted, providing more detailed information about the quality of the linkage result. This will help to make better-informed decisions about which linkage technique is best suited for a given linkage application

## Introduction

Record linkage is the process of identifying pairs or sets of records which refer to the same entity (individual) [1-4]. Traditionally, pairs of records are compared using the values in the attributes (also known as fields) common to the datasets to be linked. When these datasets contain records about individuals, then the attributes compared generally contain personal details such as names, addresses, and dates of birth. Based on the aggregated (pairwise) similarities when such attribute values are compared between records [1], the compared record pairs are considered to refer to the same individual, known as a match (if for example the resulting similarities are above a given threshold), or to two different individuals (non-match).

Unlike traditional record linkage (as commonly applied in statistics, health informatics, or computer science), the aim of group-based record linkage is to identify sets of records which either refer to the same individual or to the same group of individuals. In recent times, group-based record linkage techniques have become more popular and used in applications such as family and household identification [5,6].

For example, for the task of population reconstruction [7], one of the preliminary tasks conducted by historians, genealogists, or demographers is the identification of sibling groups, known as bundles, through the identification of birth records of children born to the same parents. Linked bundles of siblings allow a variety of studies, for example, about fertility and mortality and how these change over time [8]. Population reconstruction is an intensive, time consuming exercise due to the difficulties posed by name variations (caused by changes in spellings or the use of nicknames), temporal changes of attribute values (like change of residence over time), spelling and transcription errors, and the skewed frequency distributions of attribute values which is common in many (historical) datasets.

Traditionally, historians and genealogists have worked with birth, marriage, and death records from no more than a few parishes [9]. They are very likely to begin with a marriage which occurs within a parish and work forward in time through the birth records of that parish looking for births to that set of parents. If they see a continuing set of births, or the death of one or the other spouse they know that the family group is still within their parish. They will continue to observe the group until they determine that the woman has reached her 50th birthday, or the marriage has been brought to an end by the death of one or the other spouse. These rules followed by demographers when linking births to marriages are as described by Wrigley and colleagues [10].

Since historians are generally interested in the most certain links, they will want to know whether the links they made give a measure of the number of possible alternative links within a total population (or within a population sample), in order to get an understanding of the quality of their linked datasets. An example for possible alternative links would be the likelihood that there is more than one couple with the same name combination and the same marriage date and place, having children in the same geographical area during a similar time span (thus resulting in possible misclassification of records). Note that historians make use of transitive closure to infer links. That is, if they know that records *a* and *b* represent siblings as well as records *a* and *c*, they infer that records *b* and *b* must also be siblings. As increasingly large datasets are being linked in various domains, such manual linkage by domain experts is not feasible anymore and automated computer-based linkage methods are required.

Simple pairwise linkage methods are inadequate for group-based record linkage tasks [5] since pairs with low pairwise similarity in a group might never be captured (linkage quality can suffer from poor data quality as well [1]). Therefore, in computer science, graph-based clustering [11,12] approaches are commonly applied to tackle the problem of group-based record linkage, where a similarity graph is initially created representing records as nodes and the pairwise similarities as the weights of the edges (a connection between two nodes representing the two records being compared). A graph-based clustering technique applied on such a similarity graph aims to cluster the densely connected areas of the graph (where there are many nodes connected by edges indicating that these records belong to the same group of individuals), whereas sparsely connected or unconnected nodes represent individuals who do not belong to that particular group [12].

As automated computer-based linkage techniques are now increasingly being used across many domains [7], one crucial question is how well do such techniques perform - i.e. how accurate are the links identified by these techniques? In order to calculate a numerical linkage accuracy measure, ground-truth data in the form of true matches (pairs of records believed, with a predetermined level of certainty, to refer to the same individual) and non-matches (pairs of records believed, with a predetermined level of certainty, to refer to different individuals) need to be available.

However, the evaluation of the quality of clustering approaches for group-based record linkage is not a straightforward undertaking. The reason for this is that some predicted clusters (clusters created by a computer-based linkage) might only be partially correct (a cluster might contain some correct links and some wrong links). This can make the identification of which ground-truth cluster is represented by which predicted cluster, difficult.

As an example, when bundling birth records by the same parents, each cluster is supposed to represent the children of one mother and father. However, a clustering algorithm might generate some predicted clusters that are only partially correct. For instance, the true sibling group *{a,b,c}* might be split into two clusters *{a,b}* and *{c,d}*, where birth record *d* by other parents was wrongly linked to record *c*.

So far, most researchers working on group-based record linkage problems have adopted the traditional classification evaluation measures of *precision* and *recall* [1], which we define formally in the following section. Precision is calculated as the ratio of how many of the computer-generated (predicted) links between records are in fact true matches (i.e. seen in the ground-truth matches), while recall is calculated as the ratio of how many of the true matches were correctly predicted as matches by the computer algorithm. Both these measures, however, are based on the evaluation of links between individual records (record pairs) rather than clusters of records.

Despite the widespread use of these two measures to evaluate the quality of group-based record linkage results, obtaining the same precision and recall values for different clustering results does not necessarily reflect linkage outcomes of comparable quality. For traditional pairwise record linkage, precision and recall are suitable measures, assuming one is interested in the quality of the computer-generated links between individual records [1,13]. However, for group-based record linkage these measures do not provide detailed enough information about the predicted clusters. Let us explain this limitation of precision and recall with an example.

**Figure 1: Examples of different cluster predictions. Node colours represent the five true clusters, solid edges true matches (i.e. correctly predicted links), and dotted edges show wrong matches (incorrectly predicted links).**
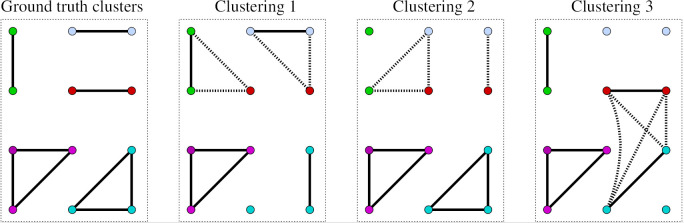


In Figure 1, we show a simple ground-truth clustering of five entity groups (clusters) in the left-most plot, and three different predicted clusterings in the other three plots. In this example, we assume the five ground-truth clusters (two made of three records and three made of two records) were created manually by an experienced genealogist whose linkage outcome can be seen to be correct with high confidence. The three clusterings 1, 2 and 3, on the other hand, are the linkage outcomes generated by three different clustering algorithms. Each ground-truth cluster in Figure 1 is represented using a different node colour. In each of the three predicted clusterings, we can see that:

- the number of true matches (correct links) is 6,
- the number of false matches (false links) is 4, and
- the number of missed true matches (missed links) is 3.

Therefore, all three of these very contrasting clustering results obtain the same precision, *P*, and recall, *R*, values of *P = 6/10 = 0.6* and *R = 6/9 = 0.667*, respectively. However, the three clusterings generated by the algorithms are all very different from one another. Depending upon the use of these linked data, for example in a public health or social science research study, one or the other of these three clusterings might be more useful. For example, a health researcher who is interested in studying siblings of larger families would likely prefer clustering 2, where two of the three clusters of size 3 are correct (whereas for clustering 1 only one of the three clusters of size 3 is correct, and for clustering 3 there is a large wrong cluster of size 4). This example shows how misleading, in the domain of group-based record linkage, the use of link-based evaluation measures such as precision and recall can sometimes be, and that the comparison of different linkage methods based on precision and recall might not be suitable.

### Contribution

To address the problem of the lack of suitable linkage evaluation measures for group-based record linkage, we propose a novel method for evaluating the quality of the clusters generated in a record linkage process, which classifies records (rather than links) according to how correctly they have been generated when compared to the ground-truth clusters. We aim to answer the question “which linkage method has generated clusters that are closest to those generated manually by an expert” (or closest to the truth in the real-world if this were available, which often is not). Since our proposed evaluation method relies on the linkage outcome alone, it is applicable for assessing any group-based record linkage method regardless of the sources being multiple datasets or a single dataset. Furthermore, this method is also suitable for assessing record linkage methods where the aim is to group records which belong to the same entity.

We acknowledge that ideally for most applications one is only interested in obtaining true links (i.e. linkage outcomes where both precision and recall are 1.0). However, as increasingly large datasets are being linked, it is necessary to employ automated computer-based linkage methods [1] since it is impossible for experts to manually link and assess millions of record pairs. Therefore, it is crucial to identify which of a selection of computer-based linkage methods produces the best linkage outcome for a given expected use of the linked data. The use of numerical evaluation measures is often the only suitable method, sometimes in combination with manual assessment of sampled record pairs or clusters, given time and resource constraints to assess the validity of linkage outcomes.

To make our work accessible to researchers and practitioners from a variety of domains, in Table 1 we show a glossary of the terms we use in this paper as well as the terms used across different domains.

**Table 1: Glossary of commonly used terms across disciplines. Each row shows terms describing the same concept as used by different fields. We highlight the terms we use in this paper in bold.** ^1^ Using birth/baptism, death/burial, and marriage records only. ^2^ Using birth/baptism, death/burial, and marriages records in conjunction with other forms of nominal records.

Relevant terms as used by different fields
**Record linkage**, data linkage, data matching, entity resolution, duplicate detection, nominal record linkage
**Group-based record linkage**, group linkage, group record linkage, record linkage of groups, similar patient matching, linkage of cohorts, family reconstitution1, family reconstruction2
**Individual**, entity
**Attribute**, field, variable, characteristic field, identifier
**False negative**, missed match, type 1 error
**False positive**, wrong match, false match, type 2 error, erroneous match
**Prediction** (of matches and non-matches), classification, processing of links

## Methods

Assuming an automated computer-based linkage of large datasets, our proposed clustering quality evaluation method considers how individual records have been allocated into predicted groups/clusters (the result of a clustering algorithm), with respect to how they appear in the ground-truth clusters. Each ground-truth cluster contains true matching records that were manually identified by a domain expert.

We follow a standard record linkage process [1,2], where we assume all records are stored in one dataset and the aim is to identify which of these records refer to the same individual or the same group of individuals. Initially, we conduct blocking on the dataset, such that records that are likely to match are grouped into the same block. Blocking helps to avoid comparing every record pair in the entire dataset, which is computationally very expensive for larger datasets [1]. All possible pairs within a block are then compared, where similarities are calculated based on the attribute (or field) values of these records. These attribute similarities are then aggregated for each record pair and normalised into 0 to 1, where a similarity of 1 reflects a perfect matching record pair (all compared attribute values are the same) while a similarity of 0 reflects a complete non-match (all compared attribute values are different). We then generate a similarity graph [14] based on these similarities, where a node represents a record in the dataset and an edge between two nodes represents the normalised aggregated similarity between the two records. An edge is created only if the aggregated similarity is greater than a user-defined minimum threshold.

We then apply a clustering technique [11,12,14] on this similarity graph. We assume the clustering technique to be a ‘black box’ in that we are not concerned with how the clustering actually works. We however assume the used clustering technique results in non-overlapping predicted clusters, where each cluster is assumed to represent one single individual or group of individuals. Some of these clusters are singletons (contain a single record) whereas others contain several records. The union of all predicted clusters contains all records in the dataset.

### Traditional link-based evaluation

To evaluate the quality of the predicted clusters, traditionally precision and recall are used where these assess the correctness of the compared record pairs [1,13]. Each record pair appearing in the same predicted cluster is considered as a positive link prediction, whereas a record pair belonging to two different predicted clusters is considered as a negative link prediction. The counts of true positive, false positive, true negative and false negative are obtained with respect to how record pairs appear in the true clusters, as shown in the error or confusion matrix [1,13] in Table 2.

The evaluation measures of precision, *P*, and recall, *R*, can be formally defined as follows [1], based on the link classification shown in Table 2:

- *P = TP / (TP+FP)* The ratio of correctly predicted links from all positive link predictions.
- *R = TP / (TP+FN)* The ratio of correctly predicted links from all true matches.

**Table 2: Confusion matrix for link classification according to ground-truth and predicted clusters.** 

		Ground-truth	
		Matches	Non-matches

Prediction	Positive Link	**True Positives (TP)** - Record pairs that appear in the same cluster both in the ground-truth and in the prediction. Known as true matches.	**False Positives (FP)** - Record pairs that appear in the same cluster in the prediction but in different clusters in the ground-truth. Known as false matches.
	Negative Link	**False Negatives (FN)** - Record pairs that appear in the same cluster in the ground-truth but in different clusters in the prediction. Known as false non-matches or missed matches.	**True Negatives (TN)** - Record pairs that appear in different clusters both in the ground-truth and prediction. Known as true non-matches.

As shown in Figure 1, precision and recall are not suitable for evaluating group-based record linkage methods that generate clusters of records because they can produce ambiguous results. They are based on the classification of links, but not of records. A user who employs several automated computer-based clustering methods and wishes to find the best such method (or the best setting of parameters when using only one linkage method) therefore cannot make a clear decision based on precision and recall only.

### Record-based cluster evaluation

To resolve this issue, our proposed method is based on classifying records instead of links for evaluation. Prior to record classification, we find the predicted cluster which best represents each ground-truth cluster. Then each record from a ground-truth cluster which appears in the corresponding best representative predicted cluster is considered a correct classification whereas the other records are considered to be misclassified. Such an evaluation is complementary to precision and recall and does not necessarily replace them. It, however, avoids the ambiguities of precision and recall.

We now describe our proposed clustering quality evaluation method in detail. Let us denote a dataset as **D** and the similarity graph as **G=(V, E)**, where **V** denotes the nodes (vertices) in the graph (all records in **D**) and **E** denotes the set of edges (the similarities calculated between records in **D**). Note that the similarity graph **G** is created based on the pairwise links resulting from the traditional pairwise record linkage approach. After applying a clustering technique on **G**, the predicted clusters contain all the records from **D**, where each cluster may be a singleton (one record) or a group of two or more records. The ground-truth clusters too may be singletons or contain several records.

We now classify each record in the ground-truth into one of seven categories, based on how they have been clustered by an algorithm. The seven categories are described in Table 3.

**Table 3: Classification of records for evaluation measures.** 

Category	Description
Correct singleton (**SS**)	These are the records which appear as singletons in both the ground-truth data and the predicted clusters.
Wrongly grouped singleton (**SG**)	These are the records which appear as singletons in the ground-truth but were assigned to a group of records in the prediction.
Missed group member (**GS**)	These are the records which appear in a group in the ground-truth, but were assigned as a singleton in the prediction.
Exact group match (**GG_E**)	These are the records contained in a predicted cluster that exactly matches a ground-truth cluster (i.e. each record in the predicted cluster appears in a ground-truth cluster, and vice versa), where the size of the cluster is larger than one.
Majority group match (**GG_M**)	A majority group match occurs when at least 50% of the records in a predicted cluster (containing at least two records) come from a single ground-truth cluster. For this classification, the best representative predicted cluster of a ground-truth cluster (which contains at least two records from the ground-truth cluster) must be identified. For a majority group match, all the records which appear in both the ground-truth cluster and predicted cluster are assigned to category **GG_M**, while all other records are classified either as **GS** or **GG_W**.
Minority group match (**GG_m**)	A minority group match is similar to a majority group match, however, less than 50% of the records in a predicted cluster come from the corresponding ground-truth cluster.
Wrongly assigned member (**GG_W**)	These are all the records from a ground-truth cluster (containing at least two records) which appear in a predicted cluster (a group) different to the majority or minority group match. That is, once we find the best representative cluster for a given ground-truth cluster, all the records which appear in a predicted cluster other than the representative cluster are assigned to this class.

This classification of records into seven categories based on their clustering can also be represented in an error or confusion matrix as shown in Table 4. The vertical columns show the true status of records (if they are a singleton or part of a cluster/group of two or more records), while the rows show the way records are predicted (again as singletons or parts of a group of records).

**Table 4: Confusion matrix for the seven categories described in Table 3:** 

	True Singleton	True Group / Cluster
Predicted Singleton	SS	GS

Predicted Group/Cluster	SG	GG_E
		GG_M
		GG_m
		GG_W

Identifying records which belong to categories **SS**, **SG**, **GS** and **GG_E** is straightforward. It can be accomplished by a single scan over the set of ground-truth clusters and the set of predicted clusters to identify all singletons in either, as well as all exactly matching groups.

However, to identify records which belong to categories **GG_M**, **GG_m**, and **GG_W**, we first require to do a mapping between ground-truth and predicted clusters such that the best representative prediction for a ground-truth cluster is identified. Subsequent to this mapping, we can identify whether each record in the ground-truth cluster appears in the correct predicted cluster or not. The reason for this requirement is that each predicted cluster can only represent one ground-truth cluster but not several. In the birth record clustering example described in Section 1, each predicted cluster can only represent the births by one mother and father; it is not possible that two predicted clusters represent the same parents.

The cluster mapping is conducted as follows. For each ground-truth cluster **gt** (which is a group) R = TP / (TP+FN) (**p_1_**, …, **p_i_**, **p_n_**) in which at least two of the records from the ground-truth cluster appear. We use the threshold value two because a predicted cluster containing just one record from the ground-truth does not contain a single true link, and is therefore inadequate to become the best representative cluster. For example, if **gt** cluster *{a,b,c}* was split as *{a}, {b}, {c}* in the prediction, it would be incorrect to identify any one of the predicted clusters to be a representation of *{a,b,c}*, because none of the true links, *a-b*, *b-c* or *a-c*, is included in the prediction. Then we calculate the similarity of gt with each predicted cluster (**p_1_**, …, **p_i_**, **p_n_**). We use the Jaccard similarity [1] and the true link similarity for this purpose, defined as:

- Jaccard similarity: ${\mathrm{sim}}_{\mathrm{Jacc}}=\frac{|\text{gt}\cap {\text{p}}_{i}|}{|\text{gt}\cup {\text{p}}_{i}|}$
  The ratio between the records common to both the ground-truth and predicted cluster, and the total number of records in the union of the two clusters. The Jaccard similarity always returns a similarity between 0 and 1.
- True link similarity: ${\mathrm{sim}}_{\mathrm{tl}}=|\text{gt}\cap {\text{p}}_{i}|$
  The number of records common to both the ground-truth and predicted cluster. This gives a positive integer similarity.

We use Jaccard similarity because of its capability of rewarding the number of records in the prediction which are from the ground-truth cluster, and penalising the records which are missed or added to the wrong predicted cluster. In an instance where the Jaccard similarities are equal for two cluster pairs, we prefer to map the cluster pair with the larger number of records first by selecting the pair with the higher true link similarity, *sim_tl_*.

Once the similarity is calculated between each ground-truth cluster, **gt**, and the corresponding predicted cluster(s), the cluster pairs are sorted in descending order of their similarities. Cluster mapping is done in a greedy manner, where the most similar clusters are mapped first. In case a ground-truth cluster is split equally into several clusters, only one is mapped to the ground-truth cluster. For example, if the ground-truth cluster *{a,b,c,d}* is split into *{a,b}* and *{c,d}*, only one of the two would be mapped to *{a,b,c,d}*. Once a ground-truth or predicted cluster is mapped, it is removed from the similarity list to ensure we obtain a one-to-one mapping where a predicted cluster represents at most one ground-truth cluster.

This process results in finding the best representative cluster **P**_*best*_ for each ground-truth cluster **gt**. However, some of the ground-truth clusters may not have a corresponding best match due to complete cluster splitting (each record in the ground-truth cluster appears in a separate cluster in the prediction) or due to an eligible predicted cluster being mapped to a different ground-truth cluster. The greedy algorithm always ensures that a ground-truth cluster is mapped to the largest predicted cluster (cluster with most matches), for as long as the corresponding predicted cluster is not already matched to another ground-truth cluster. For example, a ground-truth cluster *{a,b,c,d,e}* with predictions *{a,b,c}* and *{d,e}* is guaranteed to be mapped to the larger predicted cluster *{a,b,c}*. However, if we have two ground-truth clusters *{a,b,c,d,e}* and *{f,g,h}* with a common largest predicted cluster *{a,b,c,f,g}*, this predicted cluster would only be mapped with *{a,b,c,d,e}* because it has higher cluster similarity.

The best representative cluster is labelled as a majority or minority group match, **GG_M** or **GG_m**, based on its record composition, as described in Table 3. Once the records belonging to categories **GG_M** and **GG_m** are identified, all the records from **gt** which belong to neither of these categories, nor the **GS** category, are classified as **GG_W** (as described in Table 3).

### Example cluster evaluation

Let us illustrate our new evaluation method with an example. We will describe our proposed method with respect to the bundling (clustering) of sibling groups, where a cluster is a group of children born to the same parents. A singleton represents the only child born to parents. Each child record in the clusters predicted by an automated linkage method is classified into one of the seven categories as specified above. Even though we have used sibling clusters as an example, our proposed cluster evaluation method is applicable to any linkage technique which identifies clusters of records representing the same individual or group of individuals.

In the given example, there are five ground-truth clusters and seven predicted clusters. We will describe the classification of records with respect to each ground-truth cluster and the corresponding predicted clusters containing the records from the ground-truth cluster. In the following figures, correct links are marked with a solid line, whereas wrong links are shown with a dotted line. Furthermore, the records from the ground-truth cluster are shown in green while records that are not appearing in the ground-truth cluster are shown in red.

**Figure 2: Ground-truth cluster X.**
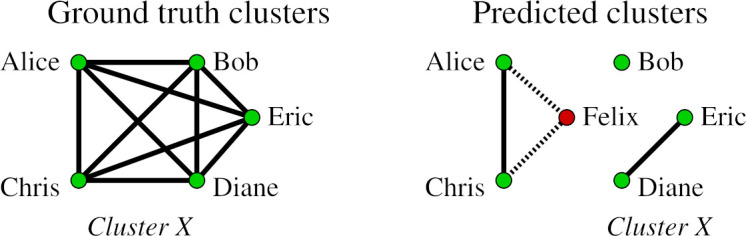


**Figure 3: Ground-truth cluster Y.**
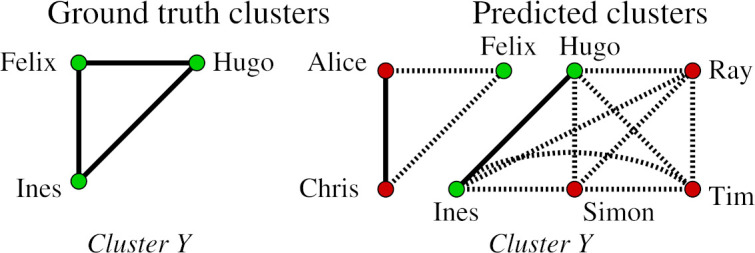


**Figure 4: Ground-truth singleton J.**
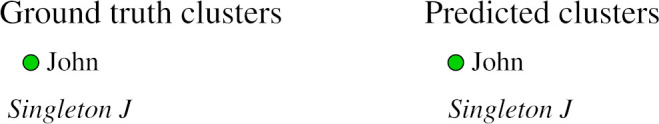


Let us consider the cluster (sibling group) containing records *{Alice,Bob,Chris,Diane,Eric}* in Figure 2. Among the predicted clusters, we initially find all the clusters where records *Alice, Bob, Chris, Diane, and Eric* appear. Among these, the ground-truth cluster *X* has the highest similarity (*sim_Jacc_* = 0.4) and with the predicted cluster *sim_tl_* = 2 with the predicted cluster *{Diane,Eric}*. Therefore, we select *{Diane,Eric}* as the best representative cluster of the sibling group *X*, and classify records *Diane* and *Eric* to be majority group matches (**GG_M**) since all records in the predicted cluster *{Diane,Eric}* come from the ground-truth cluster *X*. Record *Bob* is classified as a missed group member **(GS)** and records *Alice* and *Chris* are classified as wrongly assigned members **(GG_W)**.

Let us now consider the ground-truth cluster *{Felix,Hugo,Ines}* shown in Figure 3, which represents the sibling group *Y*. It has the highest cluster similarity with the predicted cluster *{Hugo,Ines,Ray,Simon,Tim}* (*sim_Jacc_* = 0.33 and *sim_tl_* = 2), whereas the similarity with cluster *{Alice,Chris,Felix}* is (*sim_Jacc_* = 0.2 and *sim_tl_* = 1). Therefore, the cluster *{o,Ines,Ray,Simon,Tim}* represents the sibling group *Y* in the prediction, and records *Hugo* and *Ines* are classified to be minority group matches **(GG_m)** because less than 50% of records in *{Hugo,Ines,Ray,Simon,Tim}* come from the ground-truth cluster *Y*. Record *Felix* is classified as a wrongly assigned members **(GG_W)**.

As shown in Figure 4, record John appears as a singleton both in the ground-truth and the prediction. Therefore, this record is classified as a correct singleton **(SS)**.

**Figure 5: Ground-truth singleton K:**
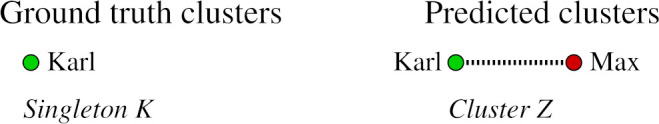


As can be seen in Figure 5, record Karl appears as a singleton in the ground-truth, but it has been assigned to a group *K = {Karl,Max}* in the prediction. Therefore, record *Karl* is classified as a wrongly grouped singleton **(SG)**.

**Figure 6: Ground-truth cluster W.**
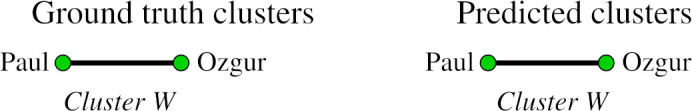


Finally, as illustrated in Figure 6, the ground-truth cluster *{Paul,Ozgur}* which represents the sibling group *W*, appears as it is in the prediction as well. Therefore, both records *Paul* and *Ozgur* are classified as exact group matches **(GG_E)**.

### Area under the curve

Most clustering algorithms have a variety of parameters that can be set by users, and based on certain settings different clusterings will be generated. One parameter common to most clustering algorithms is the minimum similarity to consider between records such that the edge between the records is included in the graph to be clustered [11,12,14,15]. As a result, for different such similarities (or different other parameter settings), different clustering outcomes for the seven categories described in Table 3 will be obtained. These different outcomes can be visualised in plots as we show in the experimental evaluation below.

**Figure 8: Plots for new evaluation results for the three clustering techniques (Names only attribute combination).**
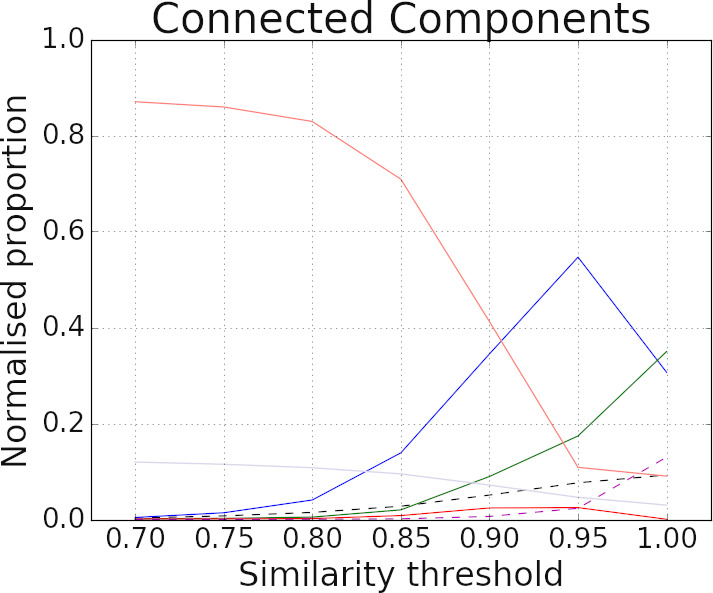

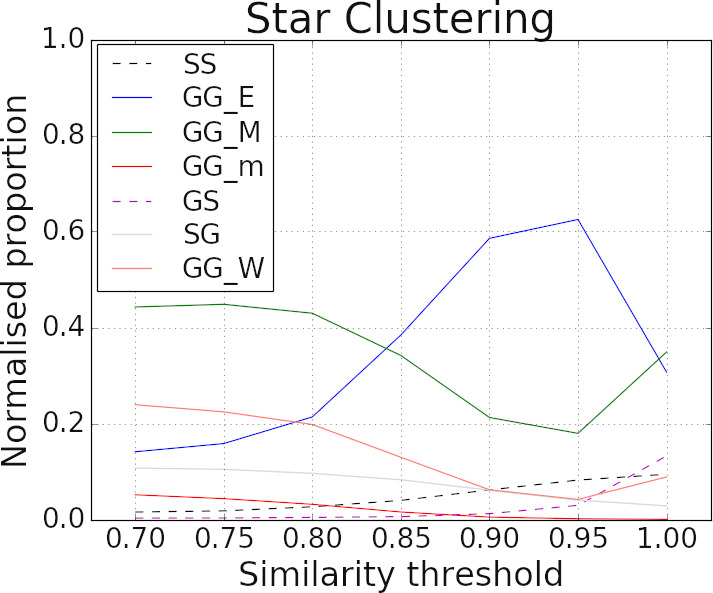

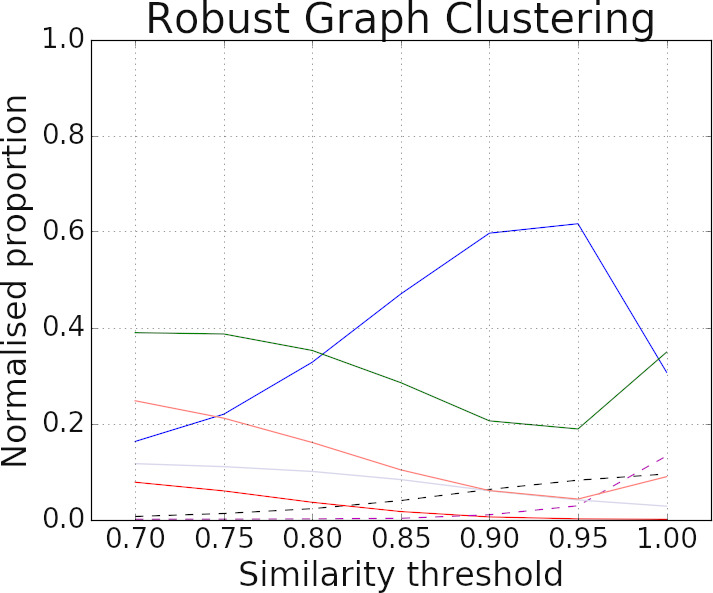
Plots for new evaluation results for the three clustering techniques *(Names only attribute combination)*.

To assess the overall performance of different clustering algorithms it is often beneficial to use a quality evaluation measure such as the Area Under the Curve (AUC) [16] to summarise linkage quality results over a range of parameter settings. We used the following approach to calculate the AUC for each of the seven categories. We plotted the normalised proportion of records which belonged to the corresponding category against the similarity threshold (as shown in Figure 8) and calculated the area under the line for each plot. Since we considered similarity thresholds ranging from 0.7 to 1.0, the sum of AUC values across the seven categories was equal to 0.3. We therefore normalised these AUC values such that the sum of AUC values resulted in 1.0.

For a better clustering approach, the AUC value of correct singleton **(SS)**, exact group match **(GG_E)**, majority group match**(GG_M)**, and minority group match **(GG_m)** should be higher whereas the values of the other categories should be lower. The differences in such AUC values allow us to describe how much better one clustering technique is over another. We illustrate the suitability of such an AUC approach using different group-based clustering techniques in the following section.

## Results

### Dataset

We conducted an experimental evaluation on a Scottish dataset that covers the population of the Isle of Skye over the period from 1861 to 1901 [8]. This dataset consists of 17,613 birth certificates, each containing personal details about a baby and its parents such as their names, addresses, occupations, and the birth date of the baby. This dataset has been linked semi-manually by demographers using Microsoft Excel and Access programmes to assist with sorting, searching, and querying records [8], and therefore ground-truth is available for conducting linkage quality evaluation. The ground-truth clusters were validated by the demographers using census, marriage, and death certificates.

**Table 5: Number of unique values and records with missing attribute values for different attributes in the Isle of Skye dataset.** 

Attribute name	Number of unique values	Number and percentage of records with a missing value
Mother’s first name	97	10 (0.06%)
Mother’s last name	286	11 (0.06%)
Father’s first name	86	955 (5.42%)
Father’s last name	301	951 (5.40%)
Mother’s occupation	73	16,446 (93.37%)
Father’s occupation	790	963 (5.47%)
Address	1,286	210 (1.19%)
Parent’s marriage date	5,105	2,346 (13.32%)

Table 5 shows the number of unique and missing values in the dataset. The frequency distribution of name attribute values was highly skewed, meaning that few names occurred many times in the dataset. However, the combination of mother’s and father’s names was relatively more distinctive compared to the other attribute combinations. Furthermore, according to demographers, the parent’s marriage dates appearing on birth certificates are not reliable. These characteristics make this dataset challenging for group-based record linkage. Note that this dataset is only used as an example for linkage, whereas any other dataset (where ground-truth data are available) is applicable for our proposed evaluation method.

### Clustering techniques

To demonstrate our record-based cluster evaluation measure, we applied three clustering algorithms1Programs and data sets are available at: https://dmm.anu.edu.au/HISTRL/ (described below) on the same pairwise similarity graph G calculated by comparing birth records using three different subsets of attributes: (1) parents’ names, their address, occupations, and marriage dates (referred to as *All* in the result figures); (2) parents’ names and addresses (referred to as *Names and addresses*); and (3) parents’ names only (referred to as *Names only*). We used weighted (referred to as *W*) and unweighted (referred to as *UW*) attribute similarities, where weights were calculated based on the Fellegi and Sunter record linkage approach [17]. Overall we generated six different similarity graphs**G**. We set the minimum similarity threshold from 0.7 to 1.0 in 0.05 steps such that only the pairwise links with at least this normalised similarity were included in the similarity graph**G**.

We incorporated temporal constraints (constraints on record linkage as implied by time differences such as a woman cannot give birth to two babies five months apart2There are however always exceptions, see: https://www.bbc.co.uk/news/world-asia-47729118)) in all three clustering approaches because previous work [14,15] has shown such constraints can improve the overall linkage quality. The three clustering techniques we have used are:

1. *Connected component clustering* considers all the pairwise links in graph **G** with a similarity greater than a user-defined similarity threshold, and then finds the connected components in the resulting graph. A connected component is a set of nodes in **G** that are directly or indirectly connected via edges. This technique has previously been used as a baseline technique to compare more sophisticated clustering approaches [11,12].
2. *Star clustering* [15] first aims to find the nodes that best represent a cluster, where these are the nodes that have the highest average similarity to their neighbouring nodes and also the highest number of neighbours in a similarity graph **G**. Cluster centers are identified iteratively, such that an unassigned node becomes a cluster center, and all its neighbours are assigned to the corresponding cluster. This process can result in overlapping clusters which are resolved by assigning a node to the cluster where it has the highest average similarity to other nodes in the cluster.
3. *Robust graph-based clustering* [14] is based on the categorisation of pairwise links according to their strength; strong, normal and weak-high (as proposed by Saeedi et al. [18]). Initially, connected components are created using a subset of link strengths, which are referred to as base clusters. Subsequently, these base clusters are iteratively merged, where the pairwise cluster similarity needs to be greater than a user-defined threshold.

### Results for the traditional precision-recall technique

**Figure 7: Precision-recall curves for three clustering techniques based on six different similarity graphs as described in Section 3 with weighted (W) and unweighted (UW) attribute similarity aggregations.**
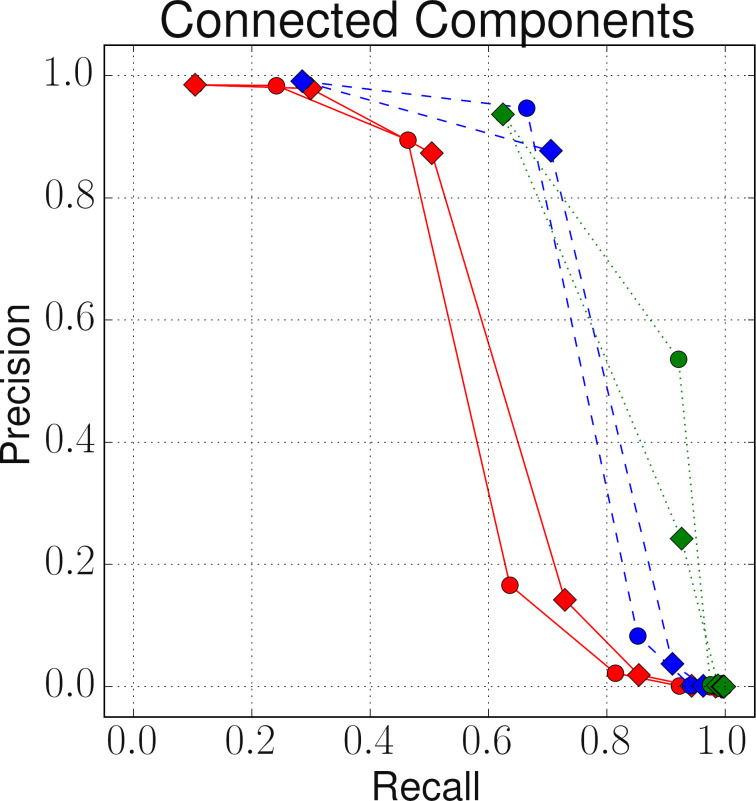

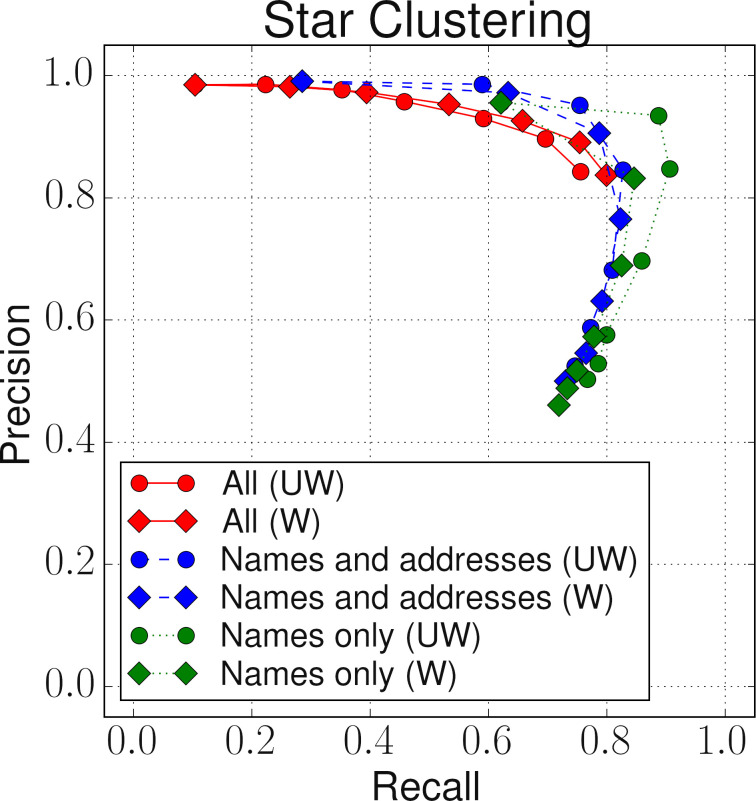

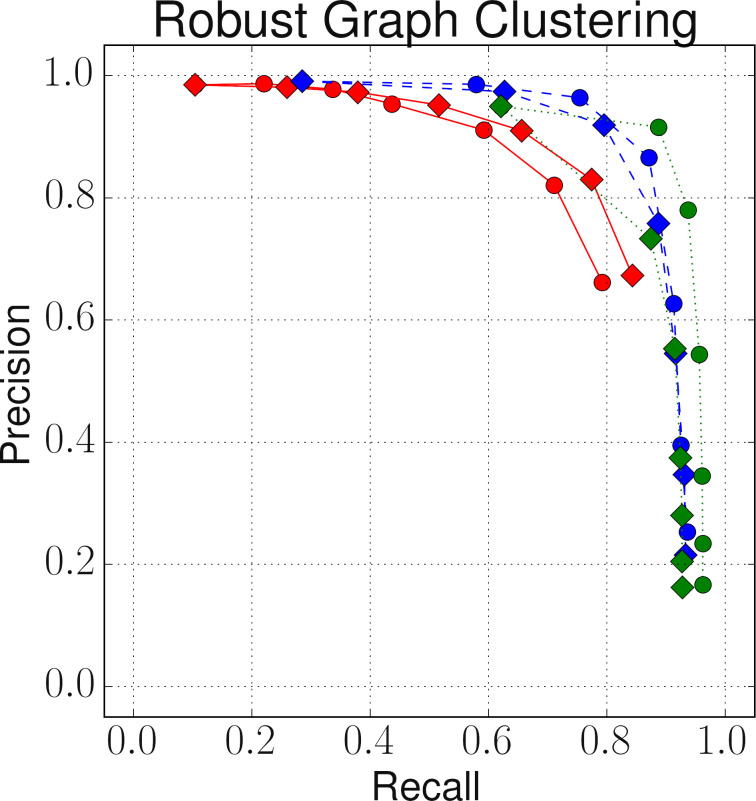


Figure 7 shows the precision-recall (PR) curves for the three clustering approaches, obtained with the best parameter settings for the six different similarity graphs. As can be seen, with all three approaches the best results were obtained when only the name attribute values (unweighted) were used to generate the similarity graph **G**. This could be attributed to missing occupation codes, incomplete marriage dates and skewness of address values as shown in Table 5. The worst linkage quality was obtained for the connected component method, whereas star clustering and robust graph clustering achieved significantly better results. Between star and robust graph clustering, the latter performed better.

### Results for our proposed evaluation method

In the following, we limit our presentation of results for our novel cluster evaluation method to only the overall best-performing *Names only (unweighted)* configuration.

Figure 8 shows the plots for our novel cluster evaluation method for the three clustering techniques. The normalised proportions of the seven categories from Table 3 are shown against the similarity threshold used for generating the similarity graph **G**. As described in Section 2, for better clustering results the values of **SS**, **GG_E**, **GG_M**, and **GG_m** should be higher whereas the values of **SG**, **GS**, and **GG_W** should be lower.

According to Figure 8, the count of **GG_M** is consistently larger than the **GG_W** count for both star and robust graph clustering approaches. The connected component technique has a much higher **GG_W** count for the majority of similarity threshold values. Furthermore, the **GG_E** count is much higher for star and robust graph clustering compared to the connected component technique. Note that the highest value of **GG_E** is obtained at the similarity threshold 0.95 for both star and robust graph clustering, which is complementary to the results shown in Figure 7. These results show that star and robust graph clustering outperform the connected component method.

**Table 6: Area under the curve (AUC) values for the three clustering techniques (Names only, unweighted attribute combination) with the best value(s) highlighted in each column.** 

Clustering technique	AUC								Average AUC for 7 categories
	PR	SS	GG_E	GG_M	GG_m	SG	GS	GG_W	

Connected components	0.744	0.036	0.206	0.077	0.01	0.087	0.017	0.567	-0.141
Star clustering	0.775	0.046	0.367	0.333	0.02	0.077	0.02	0.137	0.114
Robust graph clustering	0.885	0.044	0.413	0.298	0.027	0.077	0.017	0.124	0.123

Table 6 shows the area under the curve (AUC) results for the three clustering techniques using the best-performing *Name only (unweighted)* settings. The first column shows the AUC values for the PR curves (based on the results in Figure 7) whereas the next seven columns reflect the AUC values for our new cluster evaluation plots across the seven categories as shown in Figure 8. The last column shows a simple averaging of the AUC values across the seven categories where:

$${\mathrm{AUC}}_{\mathrm{avg}}=\frac{\left(\mathrm{SS}+{\mathrm{GG}}_{E}+{\mathrm{GG}}_{M}+{\mathrm{GG}}_{m}\right)}{4}-\frac{\left(\mathrm{SG}+\mathrm{GS}+{\mathrm{GG}}_{W}\right)}{3}$$

This average function rewards higher AUC values for categories **SS**, **GG_E**, **GG_M**, and **GG_m** and penalises high scores for the other three categories. The best values (highest for **PR**, **SS**, **GG_E**, **GG_M**, **GG_m**, and**AUC_avg_** and lowest for **SG**, **GS**, and **GG_W**) are highlighted in each of the columns in Table 6.

As can be seen from Table 6, the best **PR** and **AUC^avg^** are obtained for the robust graph clustering technique. Likewise, the highest AUC values for **GG_E** and **GG_m** are also obtained with robust graph clustering whereas the **SG**, **GS**, and **GG_W** counts are minimal with that technique. The **GG_M** and **SS** counts are highest for star clustering, whereas robust graph clustering has a slightly lower AUC for **GG_M** due to its higher count of **GG_E**. Therefore, the results shown in Table 6 further confirm our previous findings that our novel evaluation method is complementary to the PR values. Our method also provides information which is not conveyed by the PR-curve, while also being unambiguous. For example, it shows that star clustering is better than robust graph clustering for identifying singletons, due to the former having a higher **SS** count.

Based on the linkage requirement we can use weighted averaging of the seven AUC values and give a higher weight to the category or categories we want to reward more. For instance, if we are more interested in the correct identification of singletons, we can assign a higher positive weight to **SS**, higher negative weight to **SG** and relatively lower weights to the other categories. Calculating AUC values for each category is advantageous since it enables the preferential selection of linkage algorithms by weighting.

## Discussion

### Interpretation of main results

Our experimental results show that while our proposed linkage evaluation method is complementary to PR values, it also provides information that helps to select a linkage technique as required for a certain application of linked data (such as to reward identification of singletons versus groups). Furthermore, unlike with PR values, the proposed method does not provide ambiguous results. That is, the proposed method ensures that identical results are obtained for two different linkage approaches, if and only if they both have generated identical cluster predictions.

### Implications

Our analysis has shown that traditional record linkage evaluation measures such as precision and recall are inadequate for evaluating group-based record linkage due to the ambiguous results they produce. Furthermore, these traditional measures focus on evaluating the links generated in the prediction rather than assessing the assignment of records into clusters. Our novel group-based record linkage evaluation method resolves both these issues. While this is a first step towards constructing more robust linkage evaluation methods, more research is yet to be conducted in this area. Our work shows the importance of assessing linkage techniques based on several evaluation methods rather than relying on one measure. This helps to verify decisions made regarding linkage methods and understand for what purposes the linkage approaches are best suited.

### Generalisability

Even though we have presented our results using an example of sibling clustering on a single dataset, our solution is generalisable to any group-based record linkage method. That is, this method can be used to evaluate results produced by linkage techniques which group records of a single or multiple entities on single or multiple datasets. The only requirement is that the applied linkage approach produces non-overlapping groups of records. The reason for such generalisability is because our proposed method is dependent only on the ground-truth clusters and the predicted clusters.

### Strengths and limitations

As shown in the experimental evaluation, our proposed linkage evaluation method provides unambiguous results while providing insight into how the records themselves were classified; not the links between records. Furthermore, we showed that while being complementary to PR values, our novel evaluation method provides flexibility in rewarding different clustering techniques as suited for the context. As with other existing linkage evaluation methods, ours also suffers from the limitation of the requirement of ground-truth data. However, our proposed method is applicable even if the ground-truth data is incomplete. Where partial ground-truth data is available, it is possible to assess how accurately the records in the ground-truth clusters were assigned in the prediction. However, care should be taken to not tailor any ground-truth data as suited for a specific method since that could result in overfitting [1]. Rather, any ground-truth data must reflect the true state of the linkage (as best as possible) such that it allows unbiased evaluation.

## Conclusion

We have presented a novel cluster evaluation method for group-based record linkage, which, unlike the traditional measures precision and recall, does provide identical results if and only if two record linkage methods perform the same. Obtaining the same precision and recall values does not guarantee that record linkage outputs are of equal quality. In the proposed evaluation method, we classify each record in a dataset into one of seven categories to reflect how they are assigned into the predicted clusters compared to the ground-truth clusters. For comparison purposes, we propose to summarise the values obtained for the seven categories, across different parameter settings (such as varying similarity thresholds), into one area under the curve value per category.

We have illustrated our proposed cluster evaluation method on three different clustering results on a real historical dataset. This evaluation showed that the proposed method complements precision and recall and provides more detailed information regarding which aspects of a clustering algorithm is better or worse (such as whether they are more suited for correctly predicting singletons or groups). As future work, we aim to conduct the cluster mapping between ground-truth and predicted clusters using an optimal matching algorithm rather than using a greedy approach, and apply our novel evaluation method on other datasets and clustering techniques.
